# Supplementary material for: Modeling glioblastoma heterogeneity as a dynamic network of cell states
Source: Mol Syst Biol. 2021 Sep 16;17(9):e10105. doi: 10.15252/msb.202010105 (PMC8444284; doi:10.15252/msb.202010105)
Supplement: Supplementary file 6 — Source Data for Figure 5 [file MSB-17-e10105-s004.zip › Figure5A_sourcedata/GSEA_3071/hallmarks_stateIII.GseaPreranked.1623742540963/HALLMARK_ALLOGRAFT_REJECTION.html]

Details for gene set HALLMARK\_ALLOGRAFT\_REJECTION[GSEA]

|  || Dataset | state33071 |
| Phenotype | NoPhenotypeAvailable |
| Upregulated in class | na\_pos |
| GeneSet | HALLMARK\_ALLOGRAFT\_REJECTION |
| Enrichment Score (ES) | 0.23194264 |
| Normalized Enrichment Score (NES) | 1.7762352 |
| Nominal p-value | 0.0 |
| FDR q-value | 0.020153843 |
| FWER p-Value | 0.055 |
Table: GSEA Results Summary

  

Fig 1: Enrichment plot: HALLMARK\_ALLOGRAFT\_REJECTION      
 Profile of the Running ES Score & Positions of GeneSet Members on the Rank Ordered List

  

| PROBE | GENE SYMBOL | GENE\_TITLE | RANK IN GENE LIST | RANK METRIC SCORE | RUNNING ES | CORE ENRICHMENT || 1 | EIF5A |  |  | 1 | 0.691 | 0.0485 | Yes |
| 2 | RPL39 |  |  | 72 | 0.442 | 0.0617 | Yes |
| 3 | RPS3A |  |  | 82 | 0.429 | 0.0897 | Yes |
| 4 | RPS19 |  |  | 84 | 0.427 | 0.1196 | Yes |
| 5 | NPM1 |  |  | 88 | 0.421 | 0.1486 | Yes |
| 6 | EIF3J |  |  | 110 | 0.403 | 0.1716 | Yes |
| 7 | CDKN2A |  |  | 130 | 0.390 | 0.1943 | Yes |
| 8 | NME1 |  |  | 166 | 0.364 | 0.2110 | Yes |
| 9 | RPS9 |  |  | 214 | 0.331 | 0.2223 | Yes |
| 10 | RPL9 |  |  | 269 | 0.302 | 0.2298 | Yes |
| 11 | UBE2N |  |  | 339 | 0.281 | 0.2319 | Yes |
| 12 | THY1 |  |  | 472 | -0.253 | 0.2159 | No |
| 13 | ITGB2 |  |  | 514 | -0.255 | 0.2234 | No |
| 14 | ICAM1 |  |  | 568 | -0.258 | 0.2280 | No |
| 15 | GALNT1 |  |  | 704 | -0.265 | 0.2120 | No |
| 16 | TAPBP |  |  | 846 | -0.272 | 0.1950 | No |
| 17 | IL15 |  |  | 1334 | -0.297 | 0.0908 | No |
| 18 | APBB1 |  |  | 1354 | -0.298 | 0.1070 | No |
| 19 | JAK2 |  |  | 1401 | -0.300 | 0.1164 | No |
| 20 | GBP2 |  |  | 1492 | -0.306 | 0.1148 | No |
| 21 | BRCA1 |  |  | 1844 | -0.327 | 0.0477 | No |
| 22 | TIMP1 |  |  | 2038 | -0.337 | 0.0220 | No |
| 23 | TLR3 |  |  | 2059 | -0.339 | 0.0408 | No |
| 24 | IL7 |  |  | 2216 | -0.350 | 0.0254 | No |
| 25 | CTSS |  |  | 2434 | -0.368 | -0.0043 | No |
| 26 | FLNA |  |  | 2559 | -0.378 | -0.0094 | No |
| 27 | BCL3 |  |  | 2598 | -0.382 | 0.0078 | No |
| 28 | DYRK3 |  |  | 2705 | -0.391 | 0.0081 | No |
| 29 | LIF |  |  | 2908 | -0.409 | -0.0148 | No |
| 30 | CCND2 |  |  | 3037 | -0.425 | -0.0177 | No |
| 31 | ST8SIA4 |  |  | 3296 | -0.459 | -0.0516 | No |
| 32 | EGFR |  |  | 3433 | -0.488 | -0.0520 | No |
| 33 | F2R |  |  | 3538 | -0.523 | -0.0418 | No |
| 34 | CSF1 |  |  | 3669 | -0.590 | -0.0335 | No |
| 35 | TGFB2 |  |  | 3746 | -0.667 | -0.0059 | No |
| 36 | INHBA |  |  | 3801 | -0.746 | 0.0329 | No |
Table: GSEA details [plain text format]

  

Fig 2: HALLMARK\_ALLOGRAFT\_REJECTION: Random ES distribution      
 Gene set null distribution of ES for **HALLMARK\_ALLOGRAFT\_REJECTION**
